# Spatial organization of B lymphocytes and prognosis prediction in patients with gastric cancer

**DOI:** 10.1007/s10120-025-01593-y

**Published:** 2025-02-19

**Authors:** Ryan Yong Kiat Tay, Manavi Sachdeva, Haoran Ma, Young-Woo Kim, Myeong-Cherl Kook, Hyunki Kim, Jae-Ho Cheong, Lindsay C. Hewitt, Katharina Nekolla, Günter Schmidt, Takaki Yoshikawa, Takashi Oshima, Tomio Arai, Supriya Srivastava, Ming Teh, Xuewen Ong, Su Ting Tay, Taotao Sheng, Joseph J. Zhao, Patrick Tan, Heike I. Grabsch, Raghav Sundar

**Affiliations:** 1 Yong Loo Lin School of Medicine, National University of Singapore, 1E Kent Ridge Road, Singapore, 119228 Singapore; 2 Department of Haematology-Oncology, National University Cancer Institute, National University Hospital, Singapore, Singapore; 3 Cancer and Stem Cell Biology Program, Duke-NUS Medical School, Singapore, Singapore; 4 Department of Cancer Policy and Population Health, National Cancer Center Graduate School of Cancer Science and Policy and Center for Gastric Cancer and Department of Surgery, National Cancer Center, Goyang, Republic of Korea; 5 Center for Gastric Cancer, Department of Pathology, National Cancer Center, Goyang, Republic of Korea; 6 Department of Pathology, Yonsei University College of Medicine, Seoul, Republic of Korea; 7 Department of Surgery, Yonsei University College of Medicine, Seoul, Republic of Korea; 8 Department of Pathology, GROW Research Institute for Oncology and Reproduction, Maastricht University Medical Center+, Maastricht, The Netherlands; 9 Department of Precision Medicine, GROW School for Oncology and Reproduction, Maastricht University Center+, Maastricht, The Netherlands; 10 Computational Pathology, Oncology R&D, AstraZeneca, Munich, Germany; 11 Department of Surgery, Kanagawa Cancer Center, Yokohama, Japan; 12 Department of Surgery, Yokohama City University, Yokohama, Japan; 13 Department of Surgery, Tokyo Metropolitan Cancer and Infectious Diseases Center Komagome Hospital, Tokyo, Japan; 14 Department of Pathology, Tokyo Metropolitan Institute for Geriatrics and Gerontology, Tokyo, Japan; 15 Department of Medicine, National University of Singapore, Singapore, Singapore; 16 Department of Pathology, National University Hospital, Singapore, Singapore; 17 Genome Institute of Singapore, Agency for Science, Technology and Research, Singapore, Singapore; 18 Cancer Science Institute of Singapore, National University of Singapore, Singapore, Singapore; 19 Cellular and Molecular Research, National Cancer Centre, Singapore, Singapore; 20 Singhealth/Duke-NUS Institute of Precision Medicine, National Heart Centre Singapore, Singapore, Singapore; 21 Pathology and Data Analytics, Leeds Institute of Medical Research at St. James’S, University of Leeds, Leeds, UK; 22 The N.1 Institute for Health, National University of Singapore, Singapore, Singapore; 23 Singapore Gastric Cancer Consortium, Singapore, Singapore

**Keywords:** Gastric cancer, B lymphocytes, Tumor microenvironment, Immunohistochemistry, Digital Spatial Profiling

## Abstract

**Background:**

Within the tumor microenvironment (TME), the association of B lymphocytes (B cells) with prognosis and therapy response in gastric cancer (GC) remains poorly characterized. We investigated the predictive and prognostic value of B cells, including their spatial organization within the TME, in one of the largest multi-cohort studies to date.

**Methods:**

Using CD20 immunohistochemistry, we evaluated B cell density in resection specimens from 977 patients with resectable GC across three cohorts, including the randomized phase III Korean CLASSIC trial. The relationship between CD20 density, clinicopathological characteristics, and overall survival (OS) was analyzed. Digital spatial profiling of 1063 regions of interest from 15 patients was performed to characterize B cell distribution within different regions of interest (ROIs) using the NanoString GeoMx platform.

**Results:**

CD20 density was significantly higher in diffuse-type GC compared to intestinal-type (*p* = 0.000012). Patients with CD20-low diffuse-type GC had the shortest OS in the CLASSIC trial (median OS: 49 vs 62 months, HR: 1.9, 95% CI: 1.2–3.0, *p* = 0.003) and in a Japanese cohort (median OS: 49 vs 67 months, HR: 2.2, 95% CI: 1.2–4.0, *p* = 0.011). This survival difference was not seen in patients treated with adjuvant chemotherapy (median OS: 62 vs 63 months, HR: 1.8, 95% CI: 0.88–3.5, *p* = 0.108). Spatial profiling revealed significant B cell enrichment within tumor ROIs compared to the stroma, particularly in diffuse-type GC.

**Conclusions:**

Low CD20 positivity, especially in diffuse-type GC, is linked to poor prognosis and may identify patients who could benefit from chemotherapy. These findings underscore the role of B cells in GC.

**Supplementary Information:**

The online version contains supplementary material available at 10.1007/s10120-025-01593-y.

## Introduction

Gastric cancer (GC) remains the fifth most deadly cancer globally [1], despite recent advances in therapeutic regimens and a deeper understanding of its tumor microenvironment. Whilst previous studies have delineated the distribution and prognostic ability of T lymphocytes (T cells) and tumor-infiltrating lymphocytes (TILs) in its tumor microenvironment (TME) in GC [2–5], an understanding of the role of B lymphocytes (B cells) remains to be established.

Previous work using immunohistochemistry (IHC) on tissue microarrays (TMAs) found that CD20, the transmembrane antigen expressed on B cells, is associated with higher pathological risk grading, suggesting a potential role of B cells in the prognosis of patients with GC [6]. However, other studies reported opposite results with respect to the prognostic effect of CD20 [7–9]. Although these studies reported an association of CD20 expression with various clinicopathological characteristics, the effect of the interaction between CD20 expression and these clinicopathological characteristics has not been widely studied.

Other studies beyond IHC have also analyzed the association of B cells with clinicopathological characteristics [10]. B regulatory cells have been investigated previously showing an association with poorer survival in patients with GC [11]. The abundance of lymphocytes in GC from patients diagnosed with different disease stages has been studied using single cell RNA sequencing (scRNA-seq) suggesting that abundant IgA + plasma cells have been found in premalignant lesions such as chronic atrophic gastritis and intestinal metaplasia, whilst immunosuppressive myeloid and stromal cell subsets seem to dominate late-stage cancers [12]. Notably, scRNA-seq enabled lineage-based comparisons of the TME between diffuse and intestinal GC subtypes suggested increased plasma cell proportions in diffuse-type GC [13].

In summary, the current literature on the relationship between B cells and survival in GC patients remains controversial and none of the studies to date investigated the specific role of B cells in the prognosis of GC patients, as well as their association with clinicopathological characteristics. We hypothesized that B cell density has a positive effect on GC patient survival in a subset of patients with specific clinicopathological characteristics. The aim of the current study was to analyze the relationship of B cells with disease stage, histological subtypes, treatment benefit and survival in more than 1000 GC from multiple cohorts using a multi-modality approach including IHC, bulk-RNAseq and Digital Spatial Profiling (DSP).

## Materials and methods

### Clinical cohorts

#### Korean CLASSIC trial

The CLASSIC trial was a randomized, open-label, multi-center phase III study comparing D2 gastrectomy followed by adjuvant capecitabine and oxaliplatin chemotherapy with surgery alone demonstrating better survival in the adjuvant chemotherapy arm [14]. The current study was approved by the institutional review board at each participating institution and was performed in accordance with the Declaration of Helsinki and Good Clinical Practice Guidelines. All patients provided written informed consent.

#### Kanagawa Cancer Centre Hospital (KCCH) gastric cancer collection

This single hospital series from the Kanagawa Cancer Centre Hospital, Yokohama, Japan, comprises 215 cases, 89 treated with surgery alone and 126 treated with Fluorouracil-based adjuvant chemotherapy. The study was approved by the local research ethics committee.

#### Leeds Teaching Hospital NHS Trust (LTHT) gastric cancer collection

This single hospital series from the Leeds Teaching Hospitals NHS Trust, Leeds, UK, comprises 213 cases, all patients were treated by surgery alone. The use of archival tissue specimens and clinicopathological data for research had been approved by the Leeds Research Ethics Committee (CA01/122); the need for patient consent was waived by the ethics committee.

#### Stomach adenocarcinoma from The Cancer Genome Atlas (TCGA)

Transcriptomic gene expression Level 3 RSEM-normalized RNASeqV2 data and clinical data from the TCGA study of stomach adenocarcinoma (STAD) cohort were extracted from the Broad GDAC Firebrowse database [15]. The histology of all STAD TCGA samples was reviewed and the histological tumor type was classified by two pathologists from our group. Illumina HiSeq RNA-SeqV2 RSEM normalized gene values were used for B cell gene expression profile comparisons.

#### Singapore Gastric Cancer Consortium (SGCC)

For spatial transcriptomic analysis, samples from 15 patients diagnosed with GC undergoing surgical resection or endoscopic biopsy were collected at the National University Hospital (NUH), Singapore. From this cohort, 1063 unique regions of interest, identified within specific regions within the tumor microenvironment, were analyzed. This group of patients has been previously studied and detailed methods have been provided previously [16, 17]. This study was approved by the local ethics board (National Healthcare Group, Domain Specific Review Board Ref Nos: 2005/00440 and 2016/00059). Protocols were performed in accordance with the Declaration of Helsinki for Human Research.

## Experimental methods

### Immunohistochemistry

For previous studies, tissue microarrays (TMAs) were constructed from all three above-mentioned cohorts, sampling two 3 mm diameter cores (CLASSIC), two 1.2 mm diameter cores (KCCH) or three 0.6 mm cores (LTHT) from archival formalin-fixed paraffin-embedded GC resection specimens. In all cohorts, TMA cores were sampled from areas with the highest tumor density. Clinicopathological data including survival were available for all patients.

TMAs section from CLASSIC, KCCH and LTHT GC series were stained for CD20, the transmembrane antigen expressed on B cells, and other immune cell antigens such as CD3, CD8, CD31, CD45, CD66b, CD68, and CD163 as described previously [6, 14, 18–20], all slides were scanned at 40 × magnification using an Aperio scanner (Leica Microsystems, Milton Keynes, UK). For LTHT and KCCH, immunoreactive pixels per marker per core were measured using image analysis software and utilized to calculate marker density (% marker positive pixel of all pixels per core). After visual quality control with respect to tumor content and staining quality, results from cores were averaged if appropriate to establish the final value per patient.

The density of CD8 or CD20 positive pixels in the dual stained TMA sections of the CLASSIC trial cohort was estimated using a pixel-based Random Forest Classifier (Definiens Developer XD, Munich, Germany). The classifier was trained using expert annotations for CD8 positive, CD20 positive, Hematoxylin, background and artifact pixels. The percentage of positive CD8 or CD20 pixels per core was reported by the software. The same quality control as described above was performed and results from cores were averaged if appropriate to establish the final value per patient.

### Gene expression analysis of RNA-seq data

Gene expression analysis was performed on RNA-seq data from the TCGA-STAD cohort. Data were aligned to GENCODE V.19 transcript annotation using STAR v2.7.9a and TrimGalore v0.6.7. Transcripts per million abundance measure were generated using RSEM v1.3.3. RNA-seq transcripts mapping to genes profiled using the NanoString panel were extracted. Immune cell subsets were enumerated with the CIBERSORT v1.0 LM22 immune subset signature and Carcinoma EcoTyper v1.0 [21, 22]. This output yielded a set of proportions representing the estimated abundance of each immune cell type, inlcuding B cells within each sample.

### Spatial transcriptomic analysis

Digital spatial profiling (DSP) analysis was performed only on the SGCC cohort. FASTQ files from DSP were converted into count matrices using established protocols [23]. Cell abundances within each ROI were estimated using the SpatialDecon algorithm (v.1.4.3), leveraging on a human cell-profile reference matrix on Nanostring Biostats GitHub [24, 25]. Gene Set Enrichment Analysis (GSEA) of the differentially expressed genes was conducted using the MSigDB Hallmark database through the R clusterProfiler package [26]. Additional known signatures and pathways were mapped onto DSP data through single-sample GSEA (ssGSEA) using the R GSVA package.

### Statistical methods

Within the IHC cohorts, a two-sided Wilcoxon Rank Sum test was used to investigate the relationship between CD20 density and clinicopathological characteristics. Within each cohort, patients with a CD20 density greater than the 75th percentile of that cohort were classified as “CD20-high”, while patients below the 25th percentile were classified as “CD20-low”.

The cohorts were analyzed both pooled, at individual cohort level and per treatment modality. To investigate the correlation of CD20 density with other immune cell markers, the strength of correlation was measured using the Spearman (Rho, *ρ*) correlation coefficient and the probability of observing a correlation with the corresponding *p* values. A *ρ* of 0.00–0.30 was interpreted as a negligible correlation, 0.30 < *ρ* $\le$ 0.50 was interpreted as a weak correlation, 0.50 < *ρ* $\le$ 0.70 was interpreted as a moderate correlation, and *ρ* > 0.70 was interpreted as a strong correlation. Survival analyses were conducted in individual cohorts. As CLASSIC was a randomized phase III trial cohort, subgroup survival analysis by treatment was performed. Univariate survival analyses of overall survival (OS) were performed using the Kaplan–Meier method and log-rank test. Multivariate survival analyses were performed using a Cox-proportional hazards model, including all clinicopathological parameters that were significant in univariate analysis.

Within the TCGA cohort, a two-sided Wilcoxon Rank Sum test was used to investigate the relationship between B cell proportions and clinicopathological characteristics. To investigate the correlation of B cell proportions with other immune cells, the strength of correlation was measured using the Spearman (Rho, ρ) correlation coefficient and the probability of observing a correlation with the corresponding p-values. ρ was interpreted as above.

Within the DSP cohort, comparisons of B cell proportions between regions were conducted with two-sided Wilcoxon Rank Sum tests. Similar correlation analyses were performed between B cell proportions and other immune cells. All analyses were conducted in R-4.2.0 unless stated otherwise. Graphical illustrations were created with BioRender.com. A *p* value < 0.05 was considered statistically significant.

## Results

### Cohort overview

**Fig. 1:**
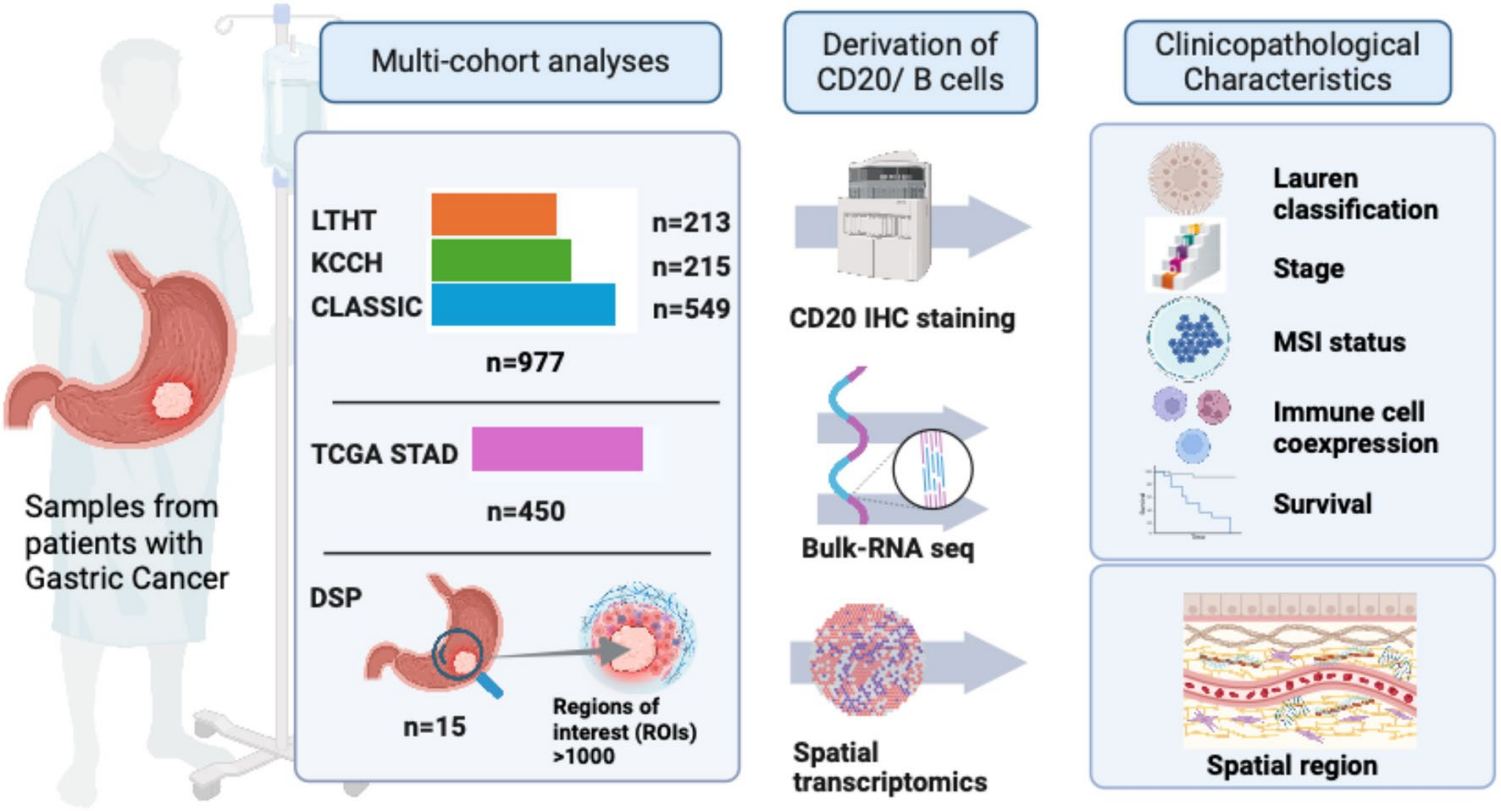
Summary of included samples including cohort details, method of CD20/B cell measurement and clinicopathological characteristics studied. Created with BioRender.com

In this study, a total of 1442 samples from multiple cohorts of patients with gastric cancer were studied using three methods: immunohistochemistry (IHC, *n* = 977), whole transcriptome sequencing (WTS, *n* = 450) and digital spatial profiling (DSP, GeoMx platform, Nanostring Technologies, Inc, *n* = 15). Among the IHC cohorts, the South Korean CLASSIC trial contributed 549 samples, the Japanese KCCH cohort 215 samples, and the UK LTHT cohort 213. An overview of the samples is provided in Fig. 1.

### CD20 density is higher in diffuse-type gastric cancer

**Fig. 2:**
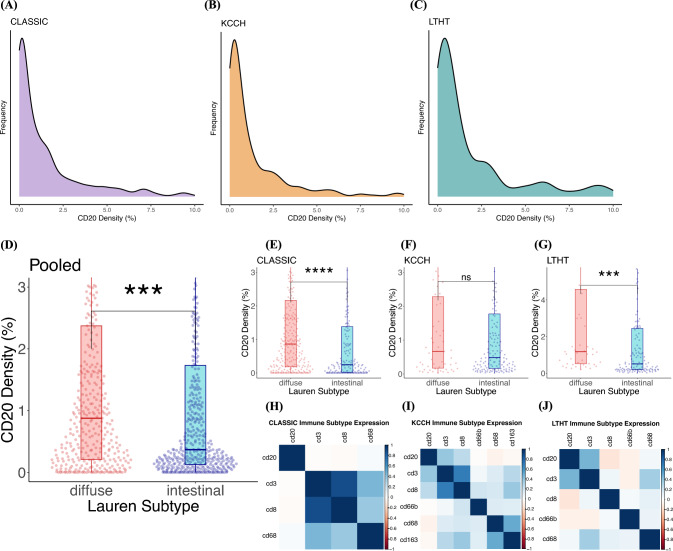
Distribution of CD20 density across cohorts, by histological subtype and correlation with other immune cell biomarkers. **A**–**C** CD20 density per cohort. **D** CD20 density is significantly higher in the diffuse-type gastric cancer compared to intestinal-type GC (pooled analysis of all cohorts). **E**–**G** Per cohort analyses confirms higher CD20 density in diffuse-type GC in the CLASSIC and LTHT cohorts, but not in the KCCH cohort. **H**–**J** Correlation of CD20 density with other immune cell biomarkers

IHC staining was performed on 977 samples across three cohorts to establish the density of CD20 positive B cells, The distribution of CD20 density (% CD20 positive pixels of all pixels per core per patient) was consistent between cohorts (Fig. 2a–c). Supplementary Table S1 summarizes the baseline clinicopathological characteristics of the samples included in this study.

When analyzed across all cohorts, CD20 density was significantly higher in diffuse-type GC (*n* = 389) compared to intestinal-type GC (*n* = 470) (1.91% vs 1.56%, *p* = 0.00025) (Fig. 2d). This association remained significant when analysing the CLASSIC trial cohort (1.82% vs. 1.25%, *p* = 0.000014) (Fig. 2e) and the LTHT cohort (2.86% vs. 1.9%, *p* = 0.0004) (Fig. 2f) individually, but was not observed in the KCCH cohort (1.58% vs. 1.64%, *p* = 0.81) (Fig. 2g). Correlation analysis indicated that CD20 density was not significantly associated with other immune cell biomarkers, such as CD3, CD8, CD31, CD45, CD66b, CD68, and CD163 (Fig. 2h–j). CD20 density was not related to any of the other clinicopathological features (Table 1).

**Table 1:** Relationship between CD20 density and clinicopathological features per cohort

		*n*	CD20 density, median %	*p* value
Histological subtype				
All cohorts	Diffuse	389	1.91	< 0.001
	Intestinal	470	1.56	
CLASSIC	Diffuse	284	1.82	< 0.001
	Intestinal	184	1.25	
LTHT	Diffuse	47	2.86	< 0.001
	Intestinal	134	1.90	
KCCH	Diffuse	58	1.56	0.81
	Intestinal	152	1.64	
Treatment modality				
CLASSIC	Surgery alone	268	1.84	0.82
	Surgery + adj. chemo	281	1.75	
KCCH	Surgery alone	89	1.67	0.51
	Surgery + adj. chemo	126	1.55	
UICC pT category				
CLASSIC	T1/T2	101	1.98	0.13
	T3/T4	448	1.75	
KCCH	T1/T2	40	2.4	0.39
	T3/T4	175	1.42	
LTHT	T1/T2	50	3.83	0.072
	T3/T4	163	1.92	
UICC pN category				
CLASSIC	N0	43	1.32	0.95
	N1 +	506	1.83	
KCCH	N0	33	1.02	0.11
	N1 +	182	1.71	
LTHT	N0	68	2.72	0.57
	N1 +	145	2.21	
Sex				
CLASSIC	Male	395	1.88	0.87
	Female	154	1.58	
KCCH	Male	157	1.42	0.094
	Female	58	2.08	
LTHT	Male	135	2.48	0.63
	Female	78	2.19	
MSI status				
CLASSIC	MSI negative	484	1.82	0.43
	MSI positive	37	1.47	
LTHT	MSI negative	106	2.15	0.034
	MSI positive	9	0.49	
KCCH	MSI negative	21	1.15	0.073
	MSI positive	192	1.66	

UICC, Union for International Cancer Control; LN, Lymph node; MSI, microsatellite instability

### Relationship of CD20 density and survival in patients with resectable gastric cancer

As expected, there were no significant differences in CD20 density in the resection specimen of patients who were treated with adjuvant chemotherapy after surgery compared to those who were treated by surgery alone (CLASSIC *p* = 0.82, KCCH *p* = 0.51). Patients with diffuse-type GC had poorer overall survival compared to those with intestinal-type GC in CLASSIC (HR 1.6; 95% CI: 1.1–2.3, *p* = 0.01)), KCCH (HR = 1.9 (95% CI = 1.3–2.9, *p* = 0.003)) and in LTHT (HR = 1.8 (95% CI = 1.2–2.6, *p* = 0.003).

**Fig. 3:**
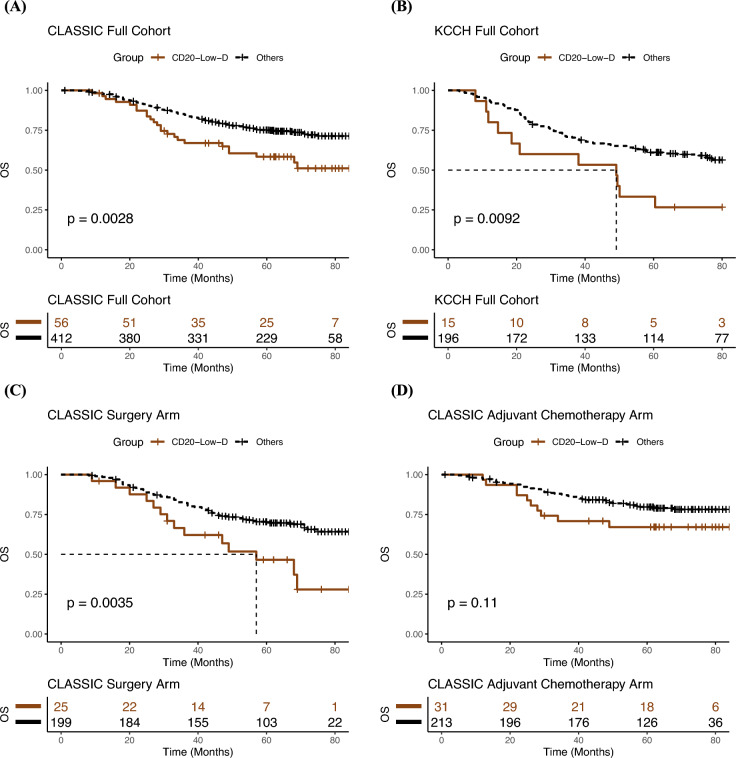
Kaplan-Meier curves depicting OS survival analysis in IHC cohorts. **A**, **B** Survival analysis shows that patients with CD20-low diffuse-type GC have the poorest prognosis in both CLASSIC and KCCH. **C** Survival analysis of the surgery alone treated patients from the CLASSIC trial shows a strong difference between CD20-low diffuse-type GC and the rest of the patients. **D** In CLASSIC, patients with CD20-low diffuse-type GC have the same survival as the rest of the patients if treated with adjuvant chemotherapy

Interestingly, patients with diffuse-type GC with low CD20 density (CD20-low diffuse-type) had the poorest OS when compared to all other patients. This was observed in patients from CLASSIC: CD20-low diffuse-type median OS = 49.0 months vs 62.0 months (HR = 1.9; 95% CI:1.2–3.0, *p* = 0.003) (Fig. 3a, Supplementary Figure S1a), and in patients from KCCH: CD20-low diffuse-type median OS = 49.1 vs 69.1 months (HR = 2.3 (95% CI = 1.2–4.2, *p* = 0.011)) (Fig. 3b, Supplementary Figure S1b). As the LTHT cohort only had 1 sample in the CD20-low diffuse-type group, this analysis could not be performed in LTHT (Supplementary Fig. S1c).

In the CLASSIC trial patients, we were able to perform further survival analyses of the CD20 density, stratifying patients by treatment. In patients treated with surgery alone, survival was significantly poorer in CD20-low diffuse-type patients (median OS = 46.0 vs 61.0 months (HR = 2.3 (95% CI = 1.3–4.2, *p* = 0.005)) (Fig. 3c, Supplementary Figure S1d). In patients treated by surgery and adjuvant chemotherapy, the difference in survival between CD20-low diffuse-type and the other patients was no longer apparent: CD20-low diffuse- type (median OS = 62.3 vs 63.0 months (HR = 1.8 (95% CI = 0.88–3.5, *p* = 0.108)) (Fig. 3d, Supplementary Figure S1e).

Multivariate analysis including CD20 density, histological subtype, combination of CD20-low and diffuse-type, disease stage, MSI status, sex, and treatment in the model, showed that while CD20 density alone was not an independent factor with respect to survival, a particular combination of CD20-low diffuse-type was associated with significantly poorer survival (Table 2). Similar univariate and multivariate analyses were also performed for the KCCH and the LTHT cohorts (Tables 3, 4).

**Table 2:** Univariate and multivariate survival analysis in the CLASSIC cohort

Variable	Univariate		Multivariate	
	HR (95% CI)	*p* value	HR (95% CI)	*p* value
CD20 density (low vs high)	0.9 (0.6–1.4)	0.8	–	–
Diffuse-type vs intestinal-type	1.6 (1.1–2.3)	0.01	–	–
CD20-low diffuse-type vs rest	1.9 (1.2–3.0)	0.003	1.8 (1.1–2.8)	0.017
Stage III vs Stage II	2.2 (1.5–3.4)	0.0003	2.4 (1.5 −3.9)	< 0.001
Tumour depth (T3/T4 vs T1/T2)	2.4 (1.3–4.5)	0.004	–	–
Nodal involvement (N1-3 vs N0)	1.2 (0.6–2.4)	0.5	–	–
Sex (male vs female)	1.6 (1.0–2.4)	0.03	1.7 (1.1–2.5)	0.015
Age (continuous)	1.0 (1.0–1.0)	0.3	–	-
MSI vs MSS	0.1 (0.015–0.8)	0.03	0.1 (0.02–0.8)	0.03
Adjuvant chemotherapy (yes vs no)	0.6 (0.4–0.9)	0.004	0.6 (0.4–0.8)	0.003

*MSI* microsatellite instability, *MSS* microsatellite stability

**Table 3:** Univariate and multivariate survival analysis in the KCCH cohort

Variable	Univariate		Multivariate	
	HR (95% CI)	*p* val	HR (95% CI)	*p* value
CD20 density (low vs high)	0.9 (0.8–1.0)	0.03	–	–
Diffuse-type vs intestinal-type	1.9 (1.3–2.9)	0.003	–	–
CD20-low diffuse-type vs rest	2.3 (1.2–4.2)	0.011	2.5 (1.3–4.7)	0.005
Stage III vs Stage II	3.3 (2.0–5.7)	< 0.001	–	–
Tumour depth (T3/T4 vs T1/T2)	2.4 (1.2–4.6)	0.001	2.9 (1.5–5.7)	0.001
Nodal involvement (N1-3 vs N0)	2.9 (1.3–6.2)	0.01	3.6 (1.7–7.9)	0.001
Sex (male vs female)	1.0 (0.6–1.6)	1.0	–	–
Age (continuous)	1.0 (1.0–1.0)	0.96	–	–
Adjuvant chemotherapy (yes vs no)	1.3 (0.8–2.0)	0.186	–	–

**Table 4:** Univariate and multivariate survival analysis in the LTHT cohort

Variable	Univariate		Multivariate	
	HR (95% CI)	*p* val	HR (95% CI)	*p* value
CD20 density (low vs high)	0.9 (0.9–1.0)	0.04	1.0 (0.9–1.0)	0.26
Diffuse-type vs intestinal-type	1.8 (1.2–2.6)	0.004	1.5 (1.0–2.2)	0.07
CD20-low diffuse-type vs rest	15.3 (2.0–116.7)	0.009	–	–
Stage III vs Stage II	2.4 (1.6–3.5)	< 0.001	–	–
Tumour depth (T3/T4 vs T1/T2)	3.9 (2.3–6.5)	< 0.001	2.2 (1.2–3.8)	**0.01**
Nodal involvement (N1-3 vs N0)	2.6 (1.7–3.8)	< 0.001	1.9 (1.2–2.9)	**0.01**
Sex (male vs female)	0.9 (0.7–1.3)	0.6	–	–
Age (continuous)	1.0 (1.0–1.0)	0.08	–	–
MSI vs MSS	0.5 (0.2–1.4)	0.2	–	–

*p* values of statistical significance were bolded

### Analysis of Bulk-RNAseq data from TCGA STAD cohort

**Fig. 4:**
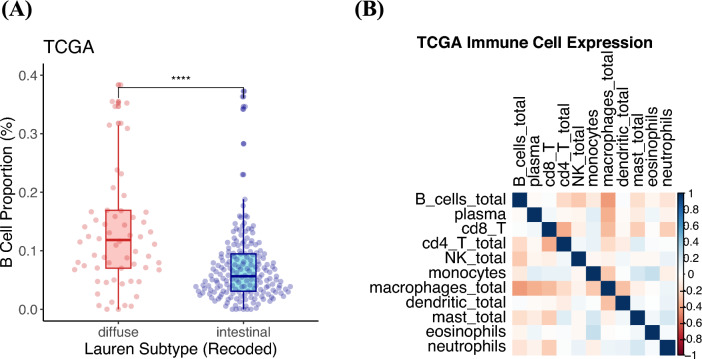
Analysis of B-cell proportions determined by Bulk RNA-seq of TCGA STAD data. **A** Similar to immunohistochemically measured CD20 density, the B-cell proportion was greater in diffuse-type GC compared to intestinal-type GC. **B** Correlation analysis with other immune cell subtypes did not find any significant correlation

To assess the generalizability of our CD20 immunohistochemical findings, Bulk-RNAseq data from TCGA (*n* = 450) was analyzed. CIBERSORT v1.0 was used to estimate immune cell proportions based on Bulk-RNAseq data. Baseline clinicopathological characteristics are outlined in Supplementary Table S2. The proportion of B cells was significantly higher in the diffuse-type GC (*n* = 66) compared to intestinal-type GC (*n* = 189) (15% vs 7%, *p* < 0.001) (Fig. 4a). The proportion of B cells was not correlated with other immune cell proportions (Fig. 4b). Supplementary Table S3 summarizes the relationship of the proportion of B cells with clinicopathological features.

### Digital spatial profiling demonstrates differences in B cell distribution within the Tumor Microenvironment

**Fig. 5:**
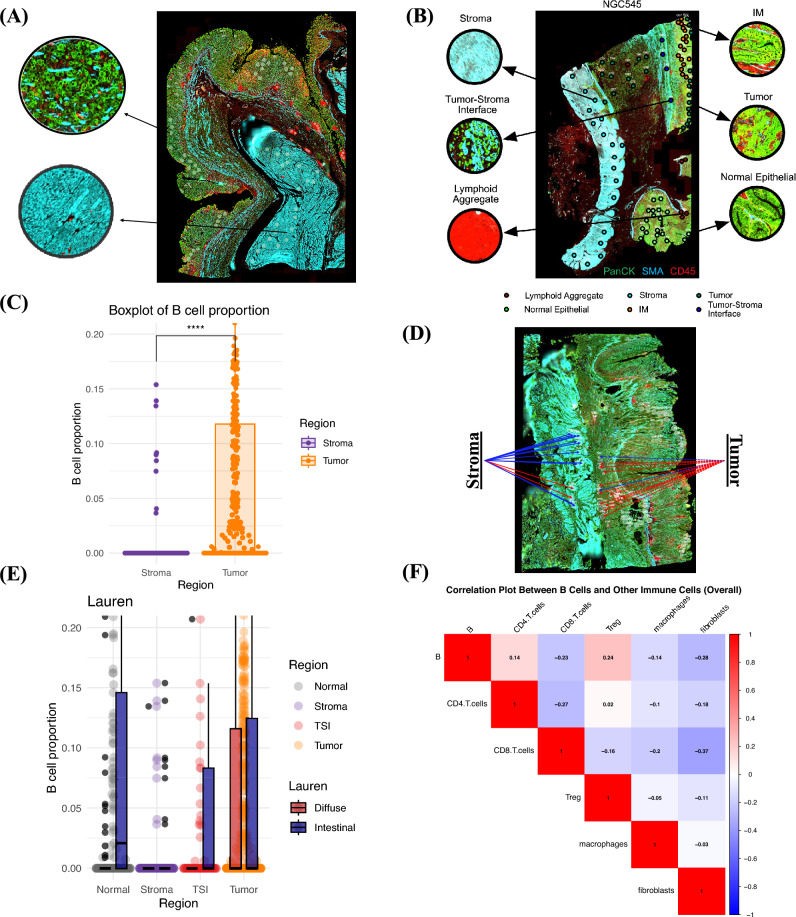
Digital spatial profiling transcriptomic analysis. **A** Haematoxylin/Eosin-stained TMA core with tumor outlined in blue to identify regions of interest (ROIs). **B** Identification of specific ROIs in one sample, NGC545. **C** Proportion of B cells in the tumor region is significantly higher than that of the intratumoral stroma region. **D** B-cell signatures are higher in tumor regions vs intratumoral stroma regions. Red lines indicate areas enriched with B cells; blue lines indicate areas with lower number of B cells. **E** B-cell distribution by region, stratified by histological subtype. Notably, diffuse-type samples appear to congregate in the tumor region, while intestinal-type samples are spread out more evenly between the other regions. **F** Immune cell proportion correlation in DSP samples does not show significant correlation with other immune cells

Exploratory analyses were conducted in 15 GC patients utilizing digital spatial profiling to establish the distribution of B cells within the TME. 1063 regions of interest (ROIs) were identified for analysis (Fig. 5a). Of these ROIs, 88 (8%) were from regions with intestinal metaplasia (IM), 130 (12%) from regions with lymphoid aggregates (LA), 179 (17%) from normal gastric epithelium, 138 (13%) from intratumoral stroma region, 11 (1%) from regions where lymphocytes were seen on top of tumor cells (TL), 87 (8%) from the tumor stromal interface (TSI) region and 430 (40%) from regions with tumor cells. A visual depiction of the staining and identification of these ROIs has been included in Fig. 5b.

B cell proportions, calculated as the % of immune cells that were B cells, within each region were compared between patients (Supplementary Figure S2a) and within the same patient (Supplementary Figure S2b). The proportion of B cells was significantly higher in the tumor region compared to the intratumoral stroma (8%vs 1%, *p* < 0.001) (Fig. 5c, d).

The distribution of the B cells within each region and its association with clinicopathological characteristics was further analyzed. In diffuse-type GC, B cells appeared to congregate in the tumor cell ROIs, while B cells were more evenly distributed between the various ROI compartments in intestinal-type GC (Fig. 5e). The proportion of B cells was significantly higher in the intestinal-type GC compared to diffuse-type GC in the normal gastric epithelium (10.8% vs 1.4%, *p* < 0.05), TSI (tumor-stromal interface) (7.3% vs 2%, *p* = 0.026), and the intratumoral stroma (1.2% vs 0.2%, *p* = 0.029) regions.

B cell proportions were also significantly higher in the tumor region in stage IV GC vs stage I-III GC (Supplementary Figure S3a) (17% vs 7%, *p* < 0.05); and higher in distal vs proximal GC (Supplementary Figure S3b) (10% vs 6%, *p* < 0.05). Correlation analysis did not find any significant correlation between B cell proportion and other immune cells (Fig. 5f) (− 0.28 < *r* < 0.24). No significant correlations were noted when stratifying the correlation analysis by histological subtype, stage or tumor location (Supplementary Figure S3c–e).

## Discussion

To date, T cells have remained the center of attention with the role of B cells in gastric cancer (GC) prediction and prognosis remaining poorly described. We aimed to characterize the role of B cells in prognosis prediction in GC patients using a multi-modality approach (immunohistochemistry, bulk-RNA sequencing and digital spatial profiling) across multiple GC cohorts. We analyzed the relationship of B cells with disease stage, histological subtypes, treatment benefit and survival in GC samples from multiple patient cohorts, including patients from the landmark phase III CLASSIC trial.

### Differences in B cell distribution by histological subtype

To understand the relationship between B cell density and GC patient prognosis, we performed one of the largest immunohistochemistry (IHC) analysis of 977 GC samples from three independent clinical cohorts and characterized the distribution of B cells in the tumor microenvironment. We demonstrated that B cell density, quantified as CD20 protein expression, was significantly greater in diffuse-type GC compared to intestinal-type gastric cancer. Orthogonal analysis of gene expression data from the TCGA STAD cohort including patients (*n* = 450) with either localized or metastatic GC further supported our findings. B cell proportion, calculated by deconvolution methods from gene expression profiles, was significantly higher in the diffuse-type GC versus intestinal-type GC suggesting that the different B cell distributions in different histological subtypes are not related to disease stage. Our CD20 immunohistochemistry and gene expression analysis results provide further evidence from a larger dataset, in support of a previous report [27].

### Survival analyses

Considering that patients with locally advanced resectable diffuse-type GC have a poor prognosis [28], and that the presence of intratumoral lymphocytes and tertiary lymphoid structures (TLS) is usually associated with a better prognosis [29], our findings of increased B cells in the diffuse-type GC appeared contradictory. We therefore performed additional exploratory survival analyses stratifying patients by CD20 density and histological subtype.

Across multiple cohorts, we found that patients with diffuse-type GC with a low CD20 (CD20-low diffuse-type) had significantly poorer survival compared to all other patients. Furthermore, we were able to leverage on data from the landmark phase III CLASSIC randomized control trial to determine whether the use of adjuvant chemotherapy has an impact on survival in this particular subgroup of patients.

The findings from our study add insight into the prognostic role of B cells in GC. Two previous GC studies using IHC suggested that CD20 positive B cell infiltration alone was associated with better prognosis [8, 9], while other IHC and bulk-RNAseq analyses found that B cell infiltration was not associated with significant survival differences [7, 10]. While the difference in prognostic outcomes may be attributed to variation in the number of patients and the methodology of the individual studies, each study also differed in terms of clinicopathological characteristics. Our study addresses this knowledge gap and is the first to demonstrate that it is a specific combination of diffuse-type samples with low CD20 that are associated with the worst prognosis. Previously proposed mechanisms may explain this observation. Studies in other cancer types highlight two ways B cells may exert an anti-tumor immune response. Firstly, via differentiation into plasma cells and subsequent antibody production, and secondly via antigen presentation to CD4 T cells within the TME [30, 31]. Considering the “immune-suppressive” features that have previously been observed in diffuse-type GC, this may explain the variability in CD20/B cell-associated survival between diffuse-type and intestinal-tpye GC [32].

Interestingly, adjuvant chemotherapy appears to be able to rescue the poor prognostic effect as patients with CD20-low diffuse-type GC randomized to adjuvant chemotherapy have the same survival as the rest of the patients in the adjuvant chemotherapy arm of the study. Chemotherapy agents are known to to induce immunogenic cell death in tumor cells [33]. Considering the role of B cells as antigen-presenting cells that mediate T cell cytotoxicity, chemotherapy may act as a compensatory mechanism in samples with lower B cell infiltration [34]. Overall, these findings provide impetus for the development of therapeutics targeting the B cell axis, perhaps particularly for patients with diffuse type GC. Significantly, we exhibit that this is show in two cohorts of distinct ethnicities and are also the first study to conduct survival analyses on samples from a randomized control trial.

### Digital spatial profiling exploratory analyses

To add further granularity to our understanding of the spatial organization of the B cells in the tumor microenvironment (TME), we performed an exploratory analysis utilizing DSP technology, analyzing more than 1000 regions of interest (ROIs) from 15 GC patients. We were able to characterize the distribution of B cell proportions within different regions in the TME and relate findings to various clinicopathological characteristics.

Our study is the first to demonstrate that B cells in GC preferably located in the tumor cell regions compared to intratumoral stromal regions. A previous pan-cancer analysis of breast, gastrointestinal and gynecological malignancies found that B cells tend to congregate in high proximity to the tumor cells [35]. Our findings confirm that this is a trend that is present specifically in GC. These results were unexpected considering that tertiary lymphoid structures, which contain B cells, are usually located in the stroma [36]. In addition, it was also interesting that we found B cells tended to congregate in the tumor cell region in diffuse-type GC, while B cells seem to be more evenly distributed across the different regions (tumor cells, normal epithelial) in intestinal-type GC. Furthermore, stage IV GC had a significantly higher proportion of B cells in the tumor cell region compared to stage I-III GC. These findings could suggest that B cells are especially present in the tumor cell region in certain histological subtypes or higher disease stages.

This study has some limitations. To date, there are no standardized cut-offs for determining what constitutes a high/low number of CD20 positive B cells in a tumor sample. Therefore, it is challenging to directly compare results between our study and previous reports on B cell density in GC and to identify the CD20 cut off most relevant for prognosis prediction and/or response to treatment. IHC samples used in our study were from early, resectable GC. This limited the feasibility of analyses on CD20/B cell density in later-stage tumors.CD20 was measured on tissue microarray cores sampled from areas with the highest tumor density irrespective of the location within the tumor. Therefore, it is not possible to assess whether there is intratumoral variation of B cell infiltration and how this might influence the predictive value of B cells. Although the spatial characterization of the GC TME highlights an important association between clinicopathological characteristics and spatial distribution of the B cells, results need to be interpreted with caution due to the relatively low number of patients in this exploratory study.

## Conclusion

To date, our study is the largest and the only multicenter cohort study including the landmark Korean CLASSIC trial of B cells in gastric cancer providing unique insights into B cell distribution and prognostic impact across multiple disease stages, histological subtypes and treatment regimens. Using a multi-modal experimental approach, our study identified in multiple cohorts that patients with diffuse-type GC containing only low levels of CD20 positive B cells have the poorest survival when treated with surgery alone. Clinically, most interesting is the finding that adjuvant chemotherapy improves the survival of this patient subgroup up to the level of the rest of the patients. We can only speculate that this effect may be attributed to the immunogenic cell death induced by the chemotherapeutic agents, which may act as a compensatory mechanism for the lower B cell infiltration, or inhibitory to the proliferative nature of diffuse-type GC. Our study is the first to describe that B cells appear to be more frequent in tumor cell regions than in the intratumoral stroma which could be relevant for the development of B cell targeting therapies. Results from our investigations highlight the important prognostic role of B cells in the GC TME, particularly in diffuse-type GC, paving the way for the development of potential therapeutics targeting the B cell axis.

## Supplementary Information

Below is the link to the electronic supplementary material.

Supplementary file1 (DOCX 1094 KB)
