# Correction: Spatial organization of B lymphocytes and prognosis prediction in patients with gastric cancer

**DOI:** 10.1007/s10120-025-01625-7

**Published:** 2025-05-16

**Authors:** Ryan Yong Kiat Tay, Manavi Sachdeva, Haoran Ma, Young-Woo Kim, Myeong-Cherl Kook, Hyunki Kim, Jae-Ho Cheong, Lindsay C. Hewitt, Katharina Nekolla, Günter Schmidt, Takaki Yoshikawa, Takashi Oshima, Tomio Arai, Supriya Srivastava, Ming Teh, Xuewen Ong, Su Ting Tay, Taotao Sheng, Joseph J. Zhao, Patrick Tan, Heike I. Grabsch, Raghav Sundar

**Affiliations:** 1 Yong Loo Lin School of Medicine, National University of Singapore, 1E Kent Ridge Road, Singapore, 119228 Singapore; 2 Department of Haematology-Oncology, National University Cancer Institute, National University Hospital, Singapore, Singapore; 3 Cancer and Stem Cell Biology Program, Duke-NUS Medical School, Singapore, Singapore; 4 Department of Cancer Policy and Population Health, National Cancer Center Graduate School of Cancer Science and Policy and Center for Gastric Cancer and Department of Surgery, National Cancer Center, Goyang, Republic of Korea; 5 Center for Gastric Cancer, Department of Pathology, National Cancer Center, Goyang, Republic of Korea; 6 Department of Pathology, Yonsei University College of Medicine, Seoul, Republic of Korea; 7 Department of Surgery, Yonsei University College of Medicine, Seoul, Republic of Korea; 8 Department of Pathology, GROW Research Institute for Oncology and Reproduction, Maastricht University Medical Center+, Maastricht, The Netherlands; 9 Department of Precision Medicine, GROW School for Oncology and Reproduction, Maastricht University Center+, Maastricht, The Netherlands; 10 Computational Pathology, Oncology R&D, AstraZeneca, Munich, Germany; 11 Department of Surgery, Kanagawa Cancer Center, Yokohama, Japan; 12 Department of Surgery, Yokohama City University, Yokohama, Japan; 13 Department of Surgery, Tokyo Metropolitan Cancer and Infectious Diseases Center Komagome Hospital, Tokyo, Japan; 14 Department of Pathology, Tokyo Metropolitan Institute for Geriatrics and Gerontology, Tokyo, Japan; 15 Department of Medicine, National University of Singapore, Singapore, Singapore; 16 Department of Pathology, National University Hospital, Singapore, Singapore; 17 Genome Institute of Singapore, Agency for Science, Technology and Research, Singapore, Singapore; 18 Cancer Science Institute of Singapore, National University of Singapore, Singapore, Singapore; 19 Cellular and Molecular Research, National Cancer Centre, Singapore, Singapore; 20 Singhealth/Duke-NUS Institute of Precision Medicine, National Heart Centre Singapore, Singapore, Singapore; 21 Pathology and Data Analytics, Leeds Institute of Medical Research at St. James’S, University of Leeds, Leeds, UK; 22 The N.1 Institute for Health, National University of Singapore, Singapore, Singapore; 23 Singapore Gastric Cancer Consortium, Singapore, Singapore

**Correction: Gastric Cancer (2025) 28:384–396** 10.1007/s10120-025-01593-y

In this article 'Katharina Nekolla' at affiliation 'Computational Pathology, Oncology R&D, AstraZeneca, Munich, Germany' was missing from the author list.

