# Supplementary material for: Spatial organization of B lymphocytes and prognosis prediction in patients with gastric cancer
Source: Gastric Cancer. 2025 Feb 19;28(3):384–96. doi: 10.1007/s10120-025-01593-y (PMC11993452; doi:10.1007/s10120-025-01593-y)
Supplement: Supplementary file 1 — Supplementary file1 (DOCX 1094 KB) [file 10120_2025_1593_MOESM1_ESM.docx]

Supplementary Material

**Supplementary Tables**

**Supplementary Table S1a:** Baseline Clinicopathological Characteristics of Samples Analysed by IHC

**Supplementary Table S1b:** Baseline Clinicopathological Characteristics of CD20-low-diffuse-type Samples Analysed by IHC

**Supplementary Table S2:** Baseline Clinicopathological Characteristics of TCGA Bulk-RNAseq samples

**Supplementary Table S3:** B Cell Proportions by clinicopathological features of TCGA Bulk-RNAseq samples

**Supplementary Table S4:** Baseline Clinicopathological Characteristics of the SGCC cohort

**Supplementary Figures**

**Supplementary Figure S1:** Kaplan-Meier curve depicting OS survival analysis in the IHC **(A)** CLASSIC cohort, **(B)** KCCH cohort and **(C)** LTHT cohort. Meaningful comparisons between CD20-Low-D samples and all other samples were not able to be made in the LTHT cohort as only one patient was identified to be in the CD20-Low-D subtype. **(D)** and **(E)** indicate the survival between groups in the CLASSIC surgery arm and adjuvant chemotherapy arm respectively.

**Supplementary Figure S2: (A)** B cell proportions across all spatial regions. **(B)** B cell proportion in each spatial region, for each sample.

**Supplementary Figure S3: (A)** Proportion of B cells in each region, split by stage **(B)** Proportion of B cells in each region, split by location **(C)** Correlation of B cells with other immune cells, between each Lauren subtype **(D)** Correlation of B cells with other immune cells, between stage I-III and stage IV samples **(E)** Correlation of B cells with other immune cells, between proximal and distal samples.

| **Supplementary Table S1a: Baseline Clinicopathological Characteristics of Samples Analysed by IHC** | | |
| --- | --- | --- |
|  | **All patients n = 977** | **%** |
| Cohort | | |
| Classic | 549 | 56.2 |
| JUST | 215 | 22.0 |
| Leeds | 213 | 21.8 |
| Lauren Subtype | | |
| Diffuse-type | 389 | 39.8 |
| Intestinal-type | 470 | 48.1 |
| Indeterminate | 106 | 10.8 |
| Unknown | 12 | 12.3 |
| Sex | | |
| Male | 687 | 70.3 |
| Female | 290 | 29.7 |
| UICC pT category | | |
| T1 – T2 | 393 | 40.2 |
| T3 – T4 | 584 | 59.8 |
| UICC pN category | | |
| N0 | 144 | 14.7 |
| N1-N3 | 833 | 85.3 |
| On Adjuvant Chemotherapy | | |
| Yes | 407 | 41.7 |
| No | 570 | 58.3 |
|  | **Mean** | **Range** |
| Age | | |
| Classic | 56.7 | 20.0-85.0 |
| JUST | 63.6 | 36.0-85.0 |
| Leeds | 70.3 | 36.55-96.0 |
|  | **Median** | **Range** |
| CD20 density (% pixels) | | |
| Classic | 0.355 | 0.00-33.0 |
| JUST | 0.615 | 0.03-18.74 |
| Leeds | 0.785 | 0.02-28.78 |

| **Supplementary Table S1b: Baseline Clinicopathological Characteristics of CD20-low-diffuse-type Samples Analysed by IHC** | | |
| --- | --- | --- |
|  | **All patients n = 72** | **%** |
| Cohort | | |
| Classic | 56 | 77.8 |
| JUST | 15 | 20.8 |
| Leeds | 1 | 1.4 |
| Lauren Subtype | | |
| Diffuse-type | 72 | 100 |
| Sex | | |
| Male | 51 | 70.8 |
| Female | 21 | 29.2 |
| UICC pT category | | |
| T1 – T2 | 6 | 8.3 |
| T3 – T4 | 66 | 91.7 |
| UICC pN category | | |
| N0 | 8 | 11.1 |
| N1-N3 | 64 | 88.9 |
| On Adjuvant Chemotherapy | | |
| Yes | 43 | 59.7 |
| No | 29 | 40.3 |
|  | **Mean** | **Range** |
| Age | | |
| Classic | 52.9 | 34.0-77.0 |
| JUST | 61.4 | 38.0-79.0 |
| Leeds | 69.3 | 69.3-69.3 |
|  | **Median** | **Range** |
| CD20 density (% pixels) | | |
| Classic | 0.0096 | 0.00-0.079 |
| JUST | 0.080 | 0.03-0.21 |
| Leeds | 0.21 | 0.21-0.21 |

| **Supplementary Table S2: Baseline Clinicopathological Characteristics of TCGA Bulk-RNAseq samples** | | |
| --- | --- | --- |
|  | **All patients n = 450** | **%** |
| Lauren Subtype | | |
| Diffuse | 66 | 14.7 |
| Intestinal | 189 | 42 |
| Mixed/Indeterminate | 16 | 35.6 |
| N/A | 179 | 39.8 |
| Sex | | |
| Male | 252 | 56 |
| Female | 139 | 30.9 |
| N/A | 59 | 13.1 |
| AJCC T stage | | |
| T1 – T2 | 105 | 23.3 |
| T3 – T4 | 277 | 61.6 |
| N/A | 68 | 15.1 |
| AJCC N stage | | |
| N0 | 117 | 26 |
| N1-N3 | 258 | 57.3 |
| N/A | 75 | 16.7 |
|  | **Mean** | **Range** |
| Age | 65.8 | 30.0-90.0 |
| B Cell Proportion | 9.45 | 0.00-62.7 |

| **Supplementary Table S3: B Cell Proportions by clinicopathological features of TCGA Bulk-RNAseq samples** | | | | |
| --- | --- | --- | --- | --- |
|  | | **B Cell Proportion (%), median** | **n** | **P-val** |
| By Lauren | | | | |
|  | Diffuse | 15 | 66 | <0.001 |
|  | Intestinal | 7 | 189 |  |
| By UICC pT category | | | | |
|  | T1/T2 | 9 | 105 | 0.0093 |
|  | T3/T4 | 10 | 277 |  |
| By Ethnicity | | | | |
|  | Asian | 11 | 81 | 0.2 |
|  | Black/African american | 9 | 10 |  |
|  | White | 10 | 245 |  |
| By Sex | | | | |
|  | Male | 9 | 252 | 0.75 |
|  | Female | 10 | 139 |  |

| **Supplementary Table S4: Baseline Clinicopathological Characteristics of the SGCC cohort** | | |
| --- | --- | --- |
|  | **All patients n = 15** | **%** |
| Lauren Subtype | | |
| Diffuse | 6 | 40.0 |
| Intestinal | 9 | 60.0 |
| Sex | | |
| Male | 9 | 60.0 |
| Female | 6 | 40.0 |
| AJCC Stage | | |
| Stage I-III | 14 | 93.3 |
| Stage IV | 1 | 6.7 |
|  | **Mean** | **Range** |
| Age | 69.7 | 36.0-86.0 |

**Supplementary Figure S1**

**Supplementary Figure S2**

**Supplementary Figure S3**
